# The economic burden of dengue fever in the Kingdom of Saudi Arabia

**DOI:** 10.1371/journal.pntd.0008847

**Published:** 2020-11-30

**Authors:** Naeema A. Akbar, Abdullah M. Assiri, Omima I. Shabouni, Osama M. Alwafi, Rajaa Al-Raddadi, Mohamad H. Alzahrani, Esam I. Azhar, Ashraf Amir, Abdullah M. Aljiffri, Abdulhakeem O. Althaqafi

**Affiliations:** 1 Preventive medicine, Public Health MOH, Jeddah, Saudi Arabia; 2 Preventive medicine, Public Health MOH, Riyadh, Saudi Arabia; 3 Preventive medicine department, Public Health MOH, Makkah, Saudi Arabia; 4 King Abdulaziz University, Faculty of Medicine, Jeddah, Saudi Arabia; 5 Special Infectious Agents Unit, King Fahd Medical Research Center & Medical Laboratory Technology Department, Faculty of Applied Medical Sciences, King Abdulaziz University, Jeddah, Saudi Arabia; 6 Chief Medical Officer, International Medical center, Jeddah, Saudi Arabia; 7 Infection Control Consultant, King Fahd Armed Forces Hospital, Jeddah, Saudi Arabia; 8 Department of medicine, King Abdulaziz Medical city- Jeddah, King Saud bin Abdulaziz university for Health Sciences, King Abdullah Medical center, Saudi Arabia; Centre hospitalier de Cayenne, FRANCE

## Abstract

Rapid urbanization, global trade, and the exceptionally great numbers of worldwide visitors during Hajj and Umrah have all placed the Kingdom of Saudi Arabia at a significant risk of introducing several vector-borne tropical diseases, such as dengue fever virus (DENV) infection. In this study we estimated DENV infection cost of illness (COI) in Saudi Arabia in the period 2013–2017, by processing national data including all declared cases recorded in referral centers in the western region, being the endemic region of the country. Using a statistically validated predictive model that was built on a representative sample of 717 laboratory-confirmed cases of DENV infection, direct costs, due to care-related expenditures, were estimated by applying the predictive equation to national data. However, indirect costs, which are due to productivity loss, were estimated using the human capital model based on gross domestic product adjusted for invalidity duration. Further, under-reporting was adjusted by using an expansion factor EF = 3. We observed highest estimated costs in 2016 with over US$168.5 Million total costs, including direct (US$29.0 Million) and indirect (US$139.5 Million) costs, for a total 4415 confirmed cases. The total DENV COI for the five years was estimated as US$551.0 Million for a total 15,369 patients (59.7%) out of 25,745 declared cases, resulting in an average cost of US$11 947.6 by patient. Depending on the year, productivity years loss costs accounted for 63.3% to 83.8% of the estimated total costs. Dengue has a substantial local economic burden that costs US$110.2 Million per year, stressing the urgent need for an effective national prevention strategy to perform considerable cost-savings besides reducing morbidity.

## Author summary

The global incidence of DENV infection has evidenced a dramatic increase in the recent two decades with a great number of cases that are misclassified or underreported. These epidemiological characteristics generate high economic costs, especially in endemic regions and countries such as Saudi Arabia. This two-phase study aimed at providing economic data that helps political efficiency and resource prioritization for dengue prevention programs, by assessing the economic burden of disease over the last five years 2013–2017. A double-method used to estimate direct costs due to care expenses and indirect costs due to productivity loss, by using a predictive and an economic model, respectively. Assuming an expansion factor of 3 to correct under-reporting, the average estimated costs of dengue illness per year in the current study was US$117.87 million. Comparison of these findings with international reports emphasized the substantial disease burden of dengue fever in Saudi Arabia. Despite some limitations, this study provided the first economic data of dengue fever infection burden in Saudi Arabia.

## Introduction

### Worldwide epidemiology of dengue

Vector-borne diseases constinue to be a major global problem, causing a significant health economic burden notably in the Eastern Mediterranean Refgions (EMR) of the World Health Organization (WHO) [1]. The global incidence of dengue virus [DENV] infection has evidenced a dramatic increase in the recent two decades and approximately 9,000 deaths are reported annually [2–5]. Therefore, the biological, epidemiological and preventive aspects of the disease have gained the attention of policy makers and funding agencies; while, the economic implication of the disease has been only recently acknowledged.

### Epidemiological and historical review of dengue fever in Saudi Arabia

In Saudi Arabia, the first case of dengue fever (DF) was reported in October 1993, in Jeddah city; and since then, a national surveillance program was implemented to monitor the disease incidence [6]. In 1995, several cities in the Western and Southern regions with marked agricultural activities and rainy conditions evidenced a DF outbreak [7]. In 2001, DF was considered to be endemic in these regions comprising the cities of Jeddah, Makkah, Madinah, and Jizan [8–11], consistent with the geographical distribution of *Aedes aegypti*, the locally identified vector of the disease [12–13].

While recent studies (2013–2017) based in Saudi Arabia provide insufficient data about DF in several regions, Jeddah is revealed to be the major contributor to increasing the total yearly number of dengue cases in the country [8,14–17]. Modeling techniques revealed that 15% of Jeddah districts were at high and 22% were at medium risk of DF [18]. Increased humidity, high temperature, and increased numbers of visitors during the annual pilgrimage (Hajj) and minor pilgrimage (Umrah) are all potential favoring conditions for disease transmission in Jeddah and the neighboring regions [9].

### Economic burden of dengue

A small number of studies estimated the global economic burden of dengue, having multiple limitations and conflicting findings; while large methodological variations are observed in regional and multinational estimates. These studies considered various expenses and used different estimation methods, data sources and economic models, which impedes the comparison of their outcomes [5,19–21]. It is expected that the quality and availability of surveillance data in several countries are based on the distribution of treatment settings and the included estimates might be based on extrapolated data. On the other hand, the economic cost of DF has no distinct profile given the variable presentation of the disease. Generally, the global economic cost of DF is likely to be higher than other major infectious diseases, including cholera, rotavirus gastroenteritis, Chagas, and canine rabies; and the main outline indicates that the burden is remarkably enormous in low-income and middle-income countries [4].

### Methods used in economic studies

Many methodological techniques can be used to investigate disease costs, including cost consequence analysis (CCA), cost minimization analysis (CMA) and cost-of-illness (COI) analysis. COI studies evaluate the direct and/or indirect costs of a disease or its risk factors. These disease-oriented approaches provide plausible and efficacious analysis in the case of dengue rather than other intervention-relevant approaches [22]. It is worthy to note that the studies that employ a COI approach may be based on prevalence-based costs (dengue-attributable costs in all individuals or a representative sample in a given year) or incidence-based costs (the values of lifetime costs of newly diagnosed patients with dengue).

As with other infectious diseases, the diagnostic and management approaches represent the main costly processes in dengue. According to recent guidelines [23], dengue has no specific medication or antiviral drug for management since the targeted drugs that act directly against DENV are still in the pipeline [24]. Therefore, treatment of symptoms remains the standard approach and hospital admission may be required, notably in intensive care, for severe cases, which may entail high costs in addition to other management aspects such as laboratory diagnosis tests and further investigations that may be necessary to rule out differential diagnoses [25–29]. Additionally, indirect costs related to loss of productivity can emerge from decreased working days of patients or their families (caregiver or companion), travel, absence from schools or activities, etc.; which may account for up to 60% to 70% of the total dengue costs, especially in cases of mortality [30–32]. However, such indirect costs may be partly underestimated or even difficult to estimate because they are often incurred by the patients or their families while other direct costs are readily available and could be provided by the relevant agencies or institutions.

### Rationale

In light of the remarkable increase in disease burden in Jeddah and the western region of Saudi Arabia and given the need to conduct more reliable studies for effective future interventions, it is becoming crucial to investigate the DF economic burden to measure its impact on both the patients and society. Quantifying such economic burden is indispensable for policymakers to allocate relevant resources, tailor specific prevention and control strategies, set priorities, and assess the cost-effectiveness of particular interventions [33–34].

### Aim & objectives

The present study aims at providing economic data that helps political efficiency and resource prioritization for dengue prevention programs, by assessing the economic burden of the disease over the period 2013–2017.

The following objectives have been achieved:

- To calculate the actual direct and indirect costs of dengue fever in a representative sample of patients;
- To analyze factors and predictors of the costs and build up a predictive model for dengue direct costs based on significant and relevant predictors;
- To estimate direct costs on national data 2013–2017 by applying the predictive models;
- To calculate indirect costs of dengue by estimating productivity loss costs using econometric models based on the gross domestic product (GDP).

## Methods

### Ethic statement

Directorate of Health Affairs—Jeddah Institutional review board (IRB) based on the good clinical practice, (GCP) guidelines. IRB Registration Number with KACST, KSA: H-020J-002. Research Number: 00712, Approval Number: A 00378, Date of approval: 3/Nov/2016. E-mail: research-jeddah@moh.gov.sa

King Fahd Armed Forces Hospital- Jeddah, research and Ethics Committee with Reference Ethical Number: REC 198. Date of approval 16/ March/2017. E-mail: mmakadi@kfafh.med.sa

Informed verbal consent was obtained from adult cases while for child cases informed verbal consent was obtained from their parents or guardians. Confidentiality and anonymity were guaranteed.

### Design, population and setting

To estimate dengue COI, this study used a Bottom-Up approach by estimating the national aggregate costs of DF based on actual costs calculated on a representative sample of patients, who were treated in one of the following centers: Ministry of Health (MOH) hospitals (King AbdulAziz Hospital and Oncology Center [KAAOC], and King Fahd General Hospital [KFGH]), King Abdulaziz University Hospital, King Fahd Armed Forces Hospital (KFAH), King Abdulaziz Medical City (KAMC), International Medical Center (IMC [private]), Bakhsh Hospital (private), Al Ahli Hospital (private). All these centers had a significant flow of patients who were registered as confirmed cases of DF during the study period.

The initial design consisted of a two-phase method for data collection and analysis, namely:

**Phase I:** calculation of actual direct and indirect costs on the representative sample of patients (**Source Database**);

**Phase II**: estimation of direct and indirect costs on national data for the years 2013–2017 (5 datasets), which was provided by the Public Health Department, at Jeddah, and is available at the Dengue Fever Operation Room Database, covering all governmental and private hospitals in Jeddah district (**Target Database**).

**Fig 1:**
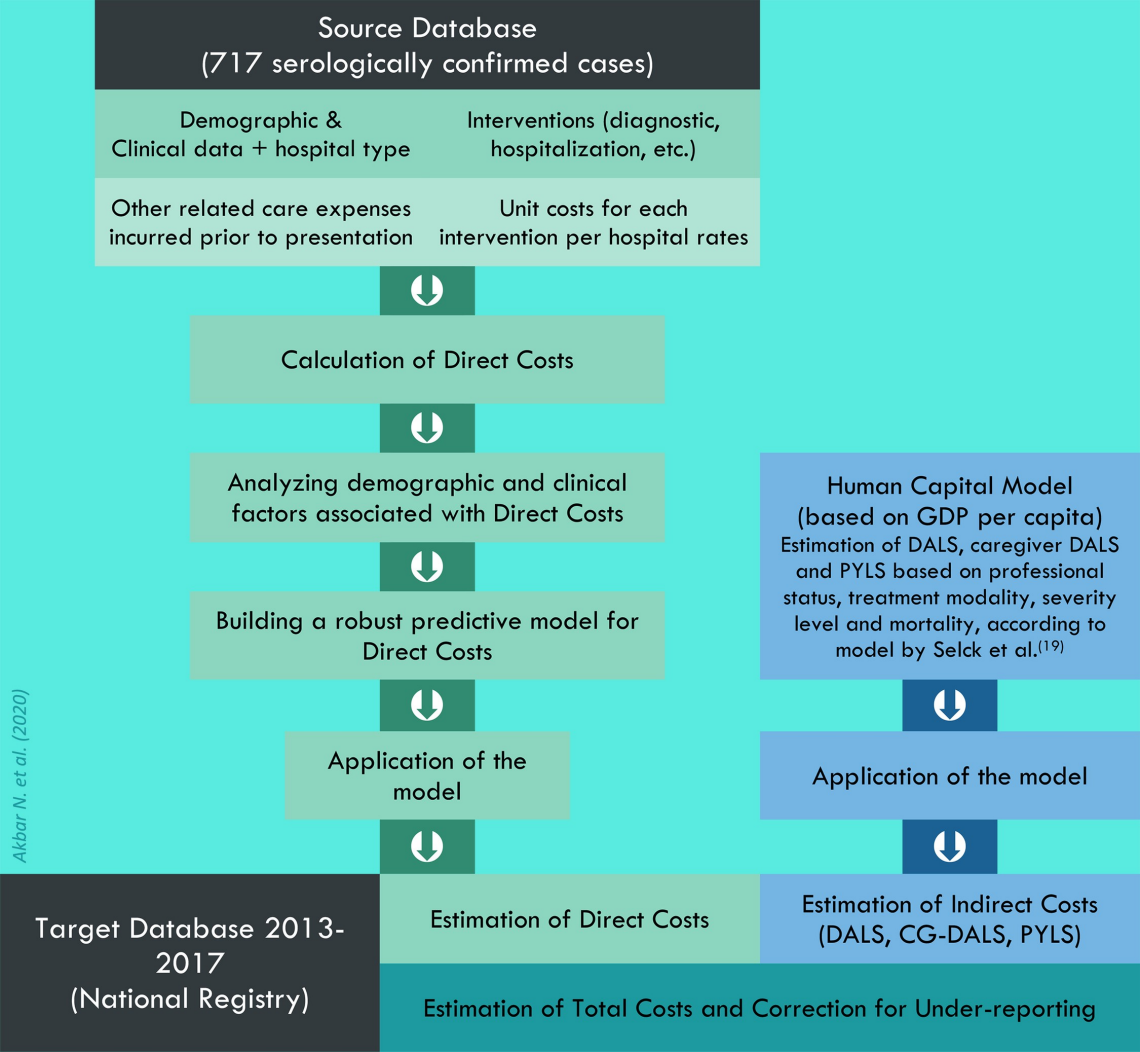
Estimation of dengue hemorrhagic fever cost of illness by estimating Direct Costs using Bottom-Up approach and Indirect Costs using Human Capital Model (Akbar et al, 2020).

However, estimation of indirect costs could not be done following this method due to substantial absence of relevant data in both Source and Target Databases. Thus, only direct costs were estimated using the two-phase Bottom-Up method; while the estimation of indirect costs used an alternative method, namely the Human Capital Model. The two methods are illustrated summarily in a flowchart (**Fig 1**) and described separately below.

### Estimation of Direct Costs (EDC) following the two-phase method

#### Phase I

A retrospective data collection was carried out to include individuals of all nationality, age and gender with laboratory-confirmed dengue (by positive polymerase chain reaction [PCR], nonstructural protein [NS] or serology), who were diagnosed and treated in 2016 in one of the selected centers (N = 717). Cases with no laboratory confirmation were excluded.

Collected data included four types of variables: 1) all health interventions (diagnostic, hospitalization, treatments, visits, etc.) provided for the patient: these data were collected by reviewing the medical records in the participating hospitals; 2) the number of units or episodes for each intervention (i.e., number of hospitalization days, clinic visits, units administered for each medication, etc.) for each patient over the management period; 3) tariffs applied for each intervention, with respect to the prices applied in each center: these data were provided by the economic officers from each hospital and completed by reviewing the Saudi Food and Drug Authority (SFDA) list of medications costs [35]; 4) sociodemographic and clinical factors that may impact direct costs: these were collected by reviewing medical records and included age, gender, marital status, nationality, professional status, disease severity level (dengue without warning signs [D.WS-], dengue with warning signs [D.WS+], and severe dengue [S.DEN.]), hospital standard (Ministry of Health [MoH] or governmental, other governmental, average-standard private, and high-standard private); in addition to hospitalization mode (ambulatory versus hospitalized) and mortality. Furthermore, participants were contacted for phone interviews to collect data on eventual out-of-pocket health expenses incurred during the same disease period, as well as any eventual past care costs including consultations, hospitalizations, medications and ER visits prior presentation to the participating center.

Direct costs of the sample (N = 717), which represent the outcome of Phase I, were calculated for each patient by summing up the unitary costs of each intervention multiplied by the number of episodes (e.g. hospitalization cost = unit cost of 1-day hospitalization * length of stay [LOS]). Specific assumptions were considered for the calculation of emergency room consultations costs (visit duration <24 hours was computed as 1 visit; >24–48 hours as 2 visits, >48–72 as 3 visits, etc.); fluid costs (if volume not specified, then 1-liter container was assumed by administration); antipyretics (if not specified, assumed to be prescribed as TID for 3 days); discharge medication (assumed to be 2-day prescription of paracetamol and omeprazole). According to the type of intervention, direct costs were divided into 4 sub-categories: a) Admissions costs (costs related to emergency room visits, hospital ward or intensive care unit stay, outpatients, etc.); b) Investigations costs (imaging and laboratory workups); c) Care costs (physician visits, nursing, fluids, medications, extra diet, etc.; and d) Past Direct Costs, corresponding to any care costs incurred prior to admission and diagnosis. Total direct costs were calculated as the sum of all intervention costs and was analyzed as the dependent variable in Phase II. Data management, processing and calculations in Phase I were done using Microsoft Excel 2013 for Windows.

#### Phase II

This Phase consisted in building the predictive model for direct costs of dengue, from the representative sample (N = 717), as a function of the relevant demographic and clinical explanatory variables including gender, age, marital status, nationality, hospital type, and severity level. This approach is based on the assumption that direct costs are independently associated with various clinical and demographic factors.

Using R Package, version 3.6.1 (Foundation for Statistical Computing, Vienna, Austria), univariate and multivariate Ordinary Least Squares (OLS) regression models were carried out to predict Direct Costs, the dependent variable, as a function of the abovementioned demographic and clinical factors. The level of statistical significance was fixed as p<0.05. The dependent variable, Direct Costs, was not normally distributed; therefore, it underwent Box-Cox transformation using a lambda = 0.1, as it was the optimal lambda value for the equation, and one that enabled the greatest Log-Likelihood. The normality of the Box-Cox transformed variable was tested by the creation of a histogram for the residuals, which indicated that the residuals are normally distributed. The OLS model using Box-Cox transformed Direct Costs was tested for other basic assumptions including linearity, homoskedasticity and inter-independence of the explanatory variables. All assumptions were fulfilled, except homoskedasticity that was violated, with Breusch-Pagan test (statistics = 191.93, p<0.001) and NCV test (statistics = 352.34, p<0.001), indicating heteroskedasticity of the model. Consequently, robust regression was carried out using Box-Cox transformed Direct Costs as the dependent variable and the aforementioned demographic and clinical factors as the explanatory variables. Two robust regression models were built. The first model (Robust Model 1) included all six explanatory variables and showed significance for only disease severity and hospital type. The second model (Robust Model 2) included only disease severity and hospital type, and both were significant. Both Robust Models 1 and 2 were tested on the Source Database and their respective mean absolute errors, mean square errors (MSEs), and Root MSEs (RMSEs) were analyzed. These showed that Robust Model 1 had a lower MAE (10466.4) compared with Robust Model 2 (10477.2), indicating better performance of Robust Model 1 in terms of absolute error. However, Robust Model 2 showed lower MSE (271991845.5 versus 278610358.6) and RMSE (16492.2 versus 16691.6), indicating higher accuracy, in terms of error metrics, of Robust Model 2 than Robust Model 1 respectively. Consequently, Robust Model 2 including severity level and hospital type as predictors, was used to predict Box-Cox transformed Direct Costs. The robust regression equation formula with the estimated parameters are presented in the results section. The predictive model was applied on Target database (national data) for each patient, and Estimated Direct Costs (EDC) were calculated by back-transforming Box-Cox Direct Costs using a specific formula (see Results section). Total EDC were calculated for each year (2013–2017) separately, by summation of EDC for all patients in the given year.

### Estimation of indirect costs (EIC) following the human capital model

This model is based on the estimation, on the Target Databases (2013–2017), of the disability days costs, which depend on both productivity days lost and GDP per capita. Productivity days loss is divided into days of activity loss (DALS) and productivity years loss (PYLS). Both DALS and PYLS were estimated using a systematic model by Shepard et al. [30] as described by Selck et al. [19]. According to this model, DALS are assumed depending on professional status (schooled or employed), treatment modality (hospitalized versus ambulatory), and severity level (Dengue without warning signs [D.WS-] or with warning signs [D.WS+] versus severe dengue [S.DEN.]); while PALS, which apply in case of disease-related mortality, are calculated as life expectancy minus age at death. For instance, as per the model, an employed individual with D.WS+ loses 6.6 versus 9.9 activity days if he is treated in ambulatory versus hospitalized mode, respectively (**Table 1**). Further, the model considers caregiver DALS, assuming the participation of a third person (relatives, friends, etc.) to provide care, assistance, and/or hospital visits to the patient, which results in absenteeism that is estimated as half the DALS of the patient. Specific assumptions were used for estimating DALS of unemployed patients including housewives, jobseekers, retired individuals and under school-age children. Subsequently, indirect costs were calculated per patient by adjusting the GDP per capita for the number of DALS (both patient’s and caregivers if applicable); and in case of death, as the sum of per capita GDP per PYLS. Total estimated indirect costs (EIC) of dengue were calculated for each year (2013–2017) using the equation: EIC = patient DALS cost + caregiver DALS cost + PYL cost. Data management, processing and calculations were done using Microsoft Excel 2013 for Windows and SPSS version 21.0 for Windows (SPSS Inc., Chicago, IL, USA).

**Table 1:** Estimation of days of activity loss and productivity years loss depending on the level of disease and treatment modality.

Mortality	No			Yes
Treatment modality	Ambulatory	Hospitalized		-
**Severity level**	D.WS+/-	D.WS+/-	S.DEN.	Any
**Days of activity loss (DALS)**				
School days loss	4.2	5.6	14.0	0.0
Work days loss	6.6	9.9	14.0	0.0
**Productivity years loss (PYLS)**	0.0	0.0	0.0	LE–age at death
**Caregiver DALS**	0.5 * DALS of patient			0.0

According to assumptions based on estimates from Shepard et al. 2011; as adapted from Selck et al. 2014 (DOI: 10.1089/vbz.2013.1528). D.WS+/-: Dengue fever with or without warning signs; S.DEN.: severe dengue; LE: life expectancy (in our case = 75 years).

### Estimation of total costs (ETC)

Estimated total costs (ETC) were computed as: ETC = EDC+EIC

Where,

EDC: are the direct costs as estimated using the predictive model

EIC: are the indirect costs as estimated using the Human Capital Model

Results were presented as the yearly (2013–2017) and total EDC, EIC and ETC. The average cost by patient (ACP) was calculated as: ACP = ETC/N, where N is the number of confirmed cases in the given year. Costs were expressed in the local currency (Saudi Riyal [SAR]) as well as in US$.

### Missing data management

Both Source and Target databases were initially managed for missing data by using appropriate imputation methods available on the Statistical Package for Social Sciences version 21.0 for Windows (SPSS Inc., Chicago, IL, USA). Missing data were of two types: missing observations for a given variable, or missing variable (unobserved variable).

1. Missing observations concerned both Source and Target databases and included age, gender, professional status, nationality, severity level and hospital type. These were imputed using multiple regression imputations to predict the missing values for each variable as a function of the other relevant variables (e.g. predicting professional status by age, gender, nationality etc.), and with respect of the local demographic figures (i.e. percentage of students in the general population, etc.). Multiple imputations were ran and analyzed, and those with figures that were inconsistent with the local figures were excluded. For numerical variables like age, where <5% data was missing, mean substitution was used after adjusting for other variables; e.g. for a single non-Saudi male with missing age data, the mean age of single Saudi males in the sample was attributed.
2. Unobserved variables concerned only Target Databases. These included treatment modality and mortality; both variables were unoserved in the national data (Target Databases). Imputation was conducted in two steps. First, each Target Database (2013–2017) was merged individually with the Source Database (N = 717), which was used as a reference dataset for the missing variables. This step was based on the assumption that populations from source (N = 717) and target datasets (2013–2017) are comparable, and thus can be analyzed in a combined dataset. Second, unobserved variables from Target Databases were treated as missing observations and imputed by using multiple or logistic regression imputation, as appropriate, as a function of relevant independent variables from the combined database. Five imputations were performed for each variable and for each combined dataset, resulting in five imputed datasets for each Target Database (2013–2017). Imputed datasets of each year were carefully analyzed, and those which showed the best consistency with the actual epidemiological figures, by reference to source database and clinical sense, were considered valid. Imputations were rejected if they were clearly inconsistent with the Source dataset with respect of the following figures: total percentage of hospitalized patients (64.0%); mortality rate (0.7%, 95%CI = 0.2%, 1.6%); hospitalization and mortality rates by severity level (58.2% and 0.3% in D.WS-, 90.7% and 0.8% in D.WS+ and 100% and 25% in S.DEN., repectively). Valid datasets (3 for each year) were used to estimate Indirect Costs by applying the Human Capital Model, and the final Indirect Costs were calculated as the average of the 3 valid estimates for each year. As to Direct Costs, they were not affected by mortality and treatment modality, as none of the two variables was included in the predictive model for Direct Costs.

### Correction for underreporting

Based on literature, an expansion factor of 3 was assumed to correct under-reporting, which corresponds to the lowest borderline of the EF range in studies from Southeast Asia and Latin America [36–39].

## Results

### Source database participants’ characteristics (N = 717)

Demographic data of the confirmed cases of DF were characterized by remarkable male predominance (male ratio = 2.20) and relatively young age (mean [SD] age = 24.70 [15.86] years), and 55.5% were national citizens. Majority of cases were D.WS-; while 16.7% were D.WS+ and 1.1% were severe dengue entailing 63.9% of hospitalizations. Mortality rate was 0.7% (95%CI = 0.2%, 1.6%). Management of the 717 cases occasioned 506 outpatient visits, 1875 days of hospitalization, and 472 days of emergency room occupation (**Table 2**).

**Table 2:** Demographic and clinical characteristics of Source database patients (N = 717).

Parameter	Category	Frequency	Percentage
*Demographic parameters*			
Age	Mean, SD	34.70	15.86
Age category (years)	0–15	72	10.0
	>15-<45	467	66.4
	≥45	178	24.8
Gender	Female	224	31.2
	Male	493	68.8
Marital status	Single	308	43.0
	Married	407	56.7
	Widow	2	0.3
Nationality	Saudi	398	55.5
	Non-Saudi	319	44.5
Residence	Jeddah	587	81.9
	Local visitor	118	16.4
	External visitor	12	1.7
*Clinical characteristics*			
Hospital	MoH	120	16.7
	Other public	262	36.5
	Private high standard	115	16.0
	Private normal standard	220	30.7
Admission via ER	Yes	414	57.8
	No	303	42.2
Treatment modality	Hospitalization	458	63.9
	Outpatient	259	36.1
Final diagnosis (severity level)	D.WS-	589	82.1
	D.WS+	120	16.7
	Severe D.	8	1.1
Mortality	Yes	5	0.7
*Other management modality data*			
Outpatient visits	(Total, average by patient)	506	0.71
Hospital stay (days)	(Total, average by patient)	1875	2.62
ER admissions (days)	(Total, average by patient)	472	0.66
ICU admissions	(Total, average by patient)	9	0.01

Results were calculated after imputation and are presented as frequencies/percentages, except if otherwise specified. SD: Standard deviation; ER: emergency room; MoH: Ministry of Health; D.WS-: dengue without warning signs; D.WS+: dengue with warning signs

### Health consumption of dengue and the related costs in Source Database

Management of the 717 DF cases entailed 506 outpatient visits (average 0.71 visit by patient, for a total cost of 137,700 SAR), 1875 days of hospitalization (average 2.62 days by patient for a total cost of 1,345,213 SAR), and 472 days of ER (average 0.66 day by patient for a total 266,775 SAR); thus, the total costs related to admissions were 1,749,688 SAR with an average 2,440.3 SAR by patient. Total cost of investigations was 5,321,530.5 SAR (average by patient = 7,421.9 SAR), of which 636,899.9 SAR for imaging and 4,684,630.6 for blood testing. The total care costs were 6,064,008.5 SAR (average 8,457.5 SAR by patient), of which 4,917,430.7 were fluids and electrolytes (6,858.3 SAR by patient). Consequently, direct costs were calculated as 13,214,717.00 SAR (18,430 SAR by patient). Health consumption and the related costs are detailed in **Table 3**.

**Table 3:** Health consumption and costs in a representative sample of confirmed dengue patients from a selection of Jeddah hospitals (Source database, N = 717).

Expenditure / Category	Consumption (Episode)		Cost (Saudi Riyal)	
	Total	Average by patient	Total	Average by patient
**Admissions**				
**Outpatient visits**	506	0.71	137,7	192,1
**Hospitalization (days)**	1875	2.62	1345213	1876,2
**ER admissions (days)**	472	0.66	266775	372,1
ICU admissions	9	0.01	137562,3	191,86
**Admissions costs**			**1749688**	**2440,3**
**Investigations**				
**Imaging**			**636899,9**	**888,3**
Abdominal US	164	0.23		
Chest XR	467	0.65		
ECG	131	0.18		
ECO	17	0.02		
CT Scan	58	0.08		
**Hematology**			**2031543,6**	**2833,4**
WBC	2699	3.76		
Platelets	2711	3.78		
HCT	2310	3.22		
RBC	1739	2.43		
CBC	2750	3.84		
PBF (peripheral blood film)	66	0.09		
ESR	52	0.07		
Serum Ferritin	44	0.06		
Blood group	74	0.10		
RFT	197	0.27		
**Biochemistry**			**1371864**	**1900,1**
Urea	1755	2.45		
Creatinine	1835	2.56		
Sodium	1793	2.50		
Potassium	1798	2.51		
Calcium	916	1.28		
Magnesium	609	0.85		
Chloride	130	0.18		
Phosphate	107	0.15		
ABG	127	0.18		
LFT	281	0.39		
AST	1533	2.14		
ALT	2053	2.86		
LDH	719	1.00		
Total protein	122	0.17		
Total bilirubin	1417	1.98		
D-bilirubin	555	0.77		
Albumin	1487	2.07		
Globulin	4	<0.00		
PTT	1906	1.53		
INR	1116	1.56		
Lipid profile	85	0.12		
Triglyceride	70	0.10		
HbA1c	59	0.08		
**Enzymology**			**102156**	**142,5**
Troponin	63	0.09		
CKMB	189	0.26		
CK total	350	0.49		
Amylase	85	0.12		
Lipase	55	0.08		
Alkaline phosphatase	125	0.17		
**Virology**			**192531**	**268,52**
HIV	196	0.27		
HBV	215	0.30		
HCV	216	0.30		
HAV	34	0.05		
**Microbiology**			**183975**	**256,5**
Urine microscopy	411	0.57		
Urine culture	279	0.39		
Stool microscopy	106	0.15		
Stool culture	98	0.14		
Blood culture	316	0.44		
Sputum culture	63	0.09		
Throat swab culture	7	0.01		
CSF culture	2	<0.00		
Septiscreen	50	0.07		
**Dengue serodiagnostic**			**528794**	**737,51**
PCR	192	0.27		
NS1	337	0.47		
IgM	494	0.69		
IgG	467	0.65		
Rapid test	172	0.24		
**Other serotests**			**273767**	**381,82**
Malaria	189	0.26		
Widal	54	0.07		
Brucella	130	0.18		
EBV	90	0.13		
CMV	74	0.10		
Monosport test	24	0.03		
CRP	241	0.34		
Influenza 1	121	0.17		
Influenza B	121	0.17		
Corona virus	65	0.09		
H1N1	2	<0.00		
Herpes	10	0.01		
Rotavirus	9	0.01		
IMN	7	0.01		
**Total investigations costs**			**5321530,5**	**7421,9**
**Care**				
Fluids & electrolytes			4917430,7	6858,3
Blood products			645300	900
Nursing care			6448	9
Physician examination			65191	90,9
Extra diet			22890	31,9
Pumps			7334	10,2
IV medications (analgesics, antipyretics, antiifectious, etc.)			395829,8	552,1
Discharge medications			3585	5
**Total care costs**			**6064008,5**	**8457,5**
**Past care costs**			**79490**	**110,9**
**Direct costs**			**13214717,00**	**18430,57**
**Indirect costs (DALS+PALS)**			**14397657,99**	**20080,42**
**Total costs**			**27612375**	**38510,98**

### Building the predictive model for estimated direct costs (EDC)

The first robust regression model, Robust Model 1, which included all six demographic and clinical factors, showed only hospital type and severity level as significant predictors of Box-Cox transformed Direct Costs. The second model, Robust Model 2, was carried out including the two significant variables. Both models are presented in **Table 4**.

**Table 4:** Robust multivariate regression model to predict Box-Cox transformed direct costs.

Model / Predictor	Category	B	t value	p-value
*Robust Model 1*				
(Intercept)	-	13.02	37.316	<0.001*
Sex	Male	0.128	0.661	.509
Age category 2	15–45	0.135	0.444	.066
Age category 3	45+	0.645	1.844	.079
Nationality	Non-Saudi	0.373	1.761	.164
Marital status 2	Married	-0.070	-0.340	.734
Marital status 3	Divorced	1.128	1.781	.075
Marital status 4	Widowed	0.880	1.747	.081
Hospital type 2	MoH/Gov.	1.421	6.324	<0.001*
Hospital type 3	Private Hight Std.	3.068	11.379	<0.001*
Hospital type 4	Other Gov.	3.942	14.464	<0.001*
Severity 2	D.WS+	1.035	4.690	<0.001*
Severity 3	D.WS-	2.021	2.597	.009*
*Robust Model 2*				
(Intercept)	-	13.623	88.805	<0.001*
Hospital type 2	MoH/Gov.	1.360	6.276	<0.001*
Hospital type 3	Private Hight Std.	2.894	11.438	<0.001*
Hospital type 4	Other Gov.	3.822	17.624	<0.001*
Severity 2	D.WS+	1.036	4.682	<0.001*
Severity 3	D.WS-	2.063	2.647	.008*

Dependent variable: Box-Cox transformed Direct costs.

B: Robust regression coefficient; MOH/Gov.: Ministry of health and governmental hospitals; STD: standard; D.WS-: dengue without warning signs; D.WS+: dengue with warning signs; SD: severe dengue; * statistically significant result (p<0.05).

According to the predictive model above, the Box-Cox transformed Direct Costs was estimated using the equation:

$$BoxCox\;Direct\;costs=13.62+a_{i}+b_{j}$$

Where,

13.62: model intercept value of Box-Cox transformed Direct Costs

*a*_*i*_: effect of hospital type on Box-Cox transformed direct costs (Private medium standard: 0 [reference], MoH/Gov: 2.834, Private High standard: 3.822, Other governmental: 1.036)

*b*_*j*_: effect of severity level on Log-transformed direct costs (D.WS-: 0 [reference], D.WS+: 1.036, S.D: 2.063)

The Estimated Direct Costs (EDC) was obtained by back transformation of Box-Cox transformed Direct Costs using the following formula:

EDC=EXP(Ln((Lambda*Box‐CoxDirectCosts)+1)/Lambda)

Where Lambda is the Box-Cox transformation factor = 0.1.

### Imputation of mortality and treatment modality

Key figures of the 5 imputations of mortality and treatment modality by severity level for each Target Database (2013–2017) are depicted in **Table 5**. These include total percentage of hospitalizations, mortality rate, and distributions of both hospitalization and mortality rates by severity level, by reference to source dataset (N = 717). These two variables are relevant for the estimation of EIC using the Human Capital Model. In general, all imputations provided total percentage of hospitalizations that is consistent with Source Database and acceptable ranges of mortality rates by respect of the 95% confidence interval of mortality (0.2%, 1.6%) in source dataset. Inconsistencies lied principally in the distribution of hospitalizations and or mortality rate by severity level, upon which the decision of retention or rejection of the given imputation was based. Note that the high variability of mortality rates in severe dengue in some imputations is due to a small number of cases; thus, imputations with high mortality rates among severe dengue cases (e.g. 70%, 7 out of 10 severe dengue cases) were considered valid.

**Table 5:** Key results of the 5 imputations of mortality and treatment modality by severity level by year.

Dataset/ imputation	Total %Hosp.	%Hosp.*Severity^1^	Total Mortality	Mortality*Severity^2^	Decision
Source (N = 717)	64	58.2 / 90.7 / 100	0.7 (95%CI = 0.2%, 1.6%)	0.3 / 0.8 / 25.0	(Reference distribution)
Target 2013					
1	63.4	58.6 / 88.0 / 21.6	0.6	0.2 / 0.6 / 37.8	Rejected
**2**	**61.6**	**55.5 / 88.2 / 100.0**	**1.3**	**0.2 / 2.2 / 78.4**	**Putative**
3	66.3	60.6 / 95.1 / 21.6	0.4	0.1 / 0.6 / 21.6	Rejected
**4**	**63.2**	**58.2 / 84.8 / 100.0**	**1.3**	**0.9 / 0.4 / 59.5**	**Putative**
**5**	**64.2**	**58.2 / 90.5 / 100.0**	**1.1**	**0.4 / 0.6 / 73.0**	**Putative**
Target 2014					
**1**	**63.0**	**56.2 / 92.7 / 100**	**0.5**	**0.2 / 0.8 / 35.7**	**Putative**
2	66.6	61.4 / 90.7 / 57.1	0.7	0.4 / 1.0 / 35.7	Rejected
3	65.4	60.2 / 89.6 / 57.1	0.5	0.2 / 0.8 / 28.6	Rejected
**4**	**62.6**	**56.1 / 91.4 / 100.0**	**1.2**	**0.3 / 4.5 / 28.6**	**Putative**
**5**	**64.0**	**58.2 / 89.4 / 100.0**	**0.5**	**0.2 / 0.5 / 42.9**	**Putative**
Target 2015					
**1**	**65.1**	**59.6 / 91.2 / 100.0**	**0.6**	**0.5 / 0.5 / 28.6**	**Putative**
2	63.4	58.4 / 87.6 / 57.1	0.5	0.5 / 0.4 / 14.3	Rejected
**3**	**63.2**	**56.9 / 93.3 / 100**	**0.7**	**0.7 / 0.3 / 14.3**	**Putative**
**4**	**65.6**	**60.5 / 89.5 / 100**	**1.1**	**1.1 / 0.3 / 35.7**	**Putative**
5	65.5	60.3 / 90.8 / 57.1	0.6	0.2 / 1.5 / 35.7	Rejected
Target 2016					
**1**	**64.9**	**59.3 / 92.3 / 100**	**0.6**	**0.5 / 0.3 / 70.0**	**Putative**
2	66.8	61.7 / 92.0 / 83.3	0.5	0.2 / 1.4 / 44.4	Rejected
**3**	**64.3**	**60.4 / 83.0 / 100**	**1.4**	**0.5 / 5.5 / 50.0**	**Putative**
4	62.1	56.4 / 89.6 / 100	0.5	0.3 / 0.8 / 22.2	Rejected
**5**	**61.0**	**56.2 / 84.5 / 100**	**1.4**	**1.5 / 0.3 / 60.0**	**Putative**
Target 2017					
**1**	**65.3**	**57.6 / 92.9 / 100**	**0.4**	**0.2 / 0.3 / 27.3**	**Putative**
**2**	**63.9**	**56.2 / 91.6 / 100**	**0.4**	**0.2 / 1.2 / 9.1**	**Putative**
3	65.2	58.1 / 91.3 / 81.8	0.6	0.4 / 0.7 / 13.6	Rejected
**4**	**64.1**	**56.3 / 92.3 / 100**	**1.0**	**0.6 / 1.8 / 22.7**	**Putative**
5	63.7	56.0 / 94.9 / 0.0	0.7	0.2 / 1.6 / 22.7	Rejected

1 Values are percentage of hospitalizations among cases of dengue without warning signs / dengue with warning signs / severe dengue, respectively; value are mortality rates among cases of dengue without warning signs / dengue with warning signs / severe dengue, respectively. %Hosp.: Percentage of hospitalizations; CI: confidence interval.

### Estimated direct, indirect and total costs

**Fig 2:**
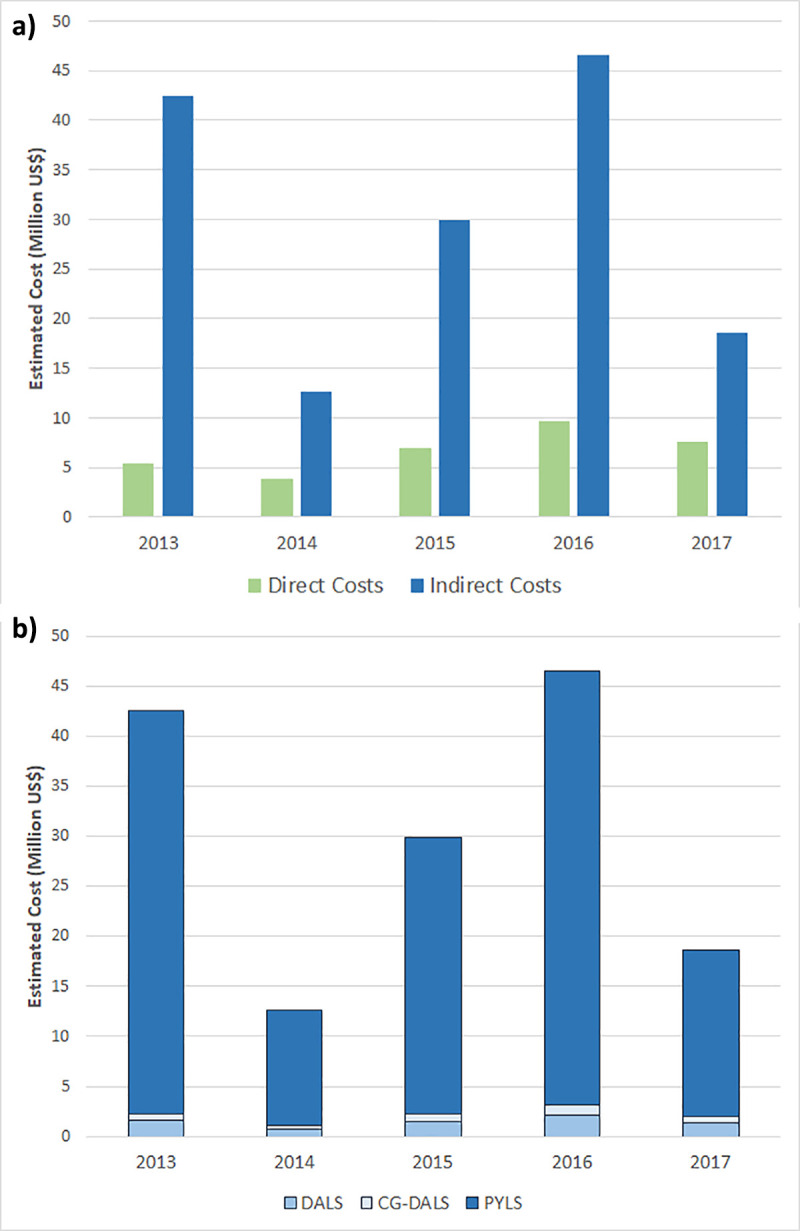
Estimated direct and indirect costs of dengue fever in Saudi Arabia during the period 2013–2017. Bars represent the amount in US$ of the (a) estimated direct and indirect costs by year, and (b) different components of indirect costs including costs related to daily activity loss (DALS), caregiver daily activity loss (CG-DALS) and productivity years loss (PYLS).

By processing imputed variables on 2013–2017 national databases, Direct Costs were calculated using the predictive model developed in Phase II analysis and Indirect Costs were calculated using the GDP-based model (Human Capital Model); final results of EDC and EIC with ETC in Saudi Riyal and US$ are presented in **Table 6**. Additionally, the 3 imputation-based calculations of indirect costs using the economic model based on GDP per capita are depicted by year and by imputation in **S1 Fig** in US$; whereas the average values of the three imputations are presented in **Table 6**. **Fig 2** depicts: **(a)** the EDC and EIC by year; and **(b)** the EIC broken up by DALS, care giver DALS (CG-DALS) and PYLS. Note that, unlike EIC, EDC do not vary by the presented imputation, as EDC did not depend on the imputed variables as per the predictive model.

**Table 6:** Estimated dengue costs in Saudi Arabia during the period 2013–2017.

Parameter	2013	2014	2015	2016	2017	5-year costs (2013–2017)
**Total No. cases**	4190	4361	4803	7219	5172	**25745**
**Confirmed**	3366	1524	3162	4415	2902	**15369**
**% Confirmed**	80.3%	34.9%	65.8%	61.2%	56.1%	**59.7%**
**EDC (SAR)**	20 406 658.5	14 324 382.7	26 252 876.3	36 226 387.78	28 301 379.8	**125 511 685.1**
**EIC (SAR)**	159 271 688.8	47 401 853.8	112 181 470.0	174 443 401.3	69 772 193.75	**563 070 607.5**
**ETC (SAR)**	179 678 347.3	61 726 236.5	138 434 346.3	210 669 789.1	98 073 573.6	**688 582 292.7**
**ETC (US$)**	47 914 225.9	16 460 329.7	36 915 825.7	56 178 610.4	26152952.9	**183 621 944.7**
**Average by patient SAR**	53 380.4	40 502.8	43 780.6	47 716.8	33 795.2	44 803.3
**Average by patient US$**	14 234.8	10 800.7	11 674.8	12 724.5	9 012.0	11 947.6

EDC: Estimated direct costs using the predictive model based on source data; EIC: estimated indirect costs using the GDP-based model; ETC: Estimated total costs; SAR: Saudi Riyal

We observed highest ETC in 2016 (210 669 789.1 SAR = 56 178 610.4 US$) for a total 4415 confirmed cases (61.2%) out of a total 7219 suspected ones; while the lowest ETC was observed in 2014 (61 726 236.5 SAR = 16 460 329.7 US$) corresponding to 1524 confirmed cases (34.9% of the 4361 suspected one). The total dengue costs for the five-year period was estimated as 688 582 292.7SAR (US$183 621 944.7), for a total 15,369 patients out of 25,745 suspected cases, resulting in an average cost of 44 803.3 SAR (US$ 11 947.6) by patient. Remarkably, EIC were substantially higher than EDC (**Fig 2A**), and, outstandingly, PYLS represented the major proportion of EIC (**Fig 2B**). Thus, PYLS accounted for 63.3% to 83.8% of the estimated total costs, depending on the year.

### Correction for under-reporting

Under-reporting was assumed as follows: for every reported dengue case, there are 3 actual dengue cases (i.e., an expansion factor of 3); thus, underreporting was corrected by multiplying by expansion factors (EF3) to scale reported cases. Results of costs after under-reporting correction are presented in **Table 7**. Thus, the yearly economic burden of dengue fever in Saudi Arabia was estimated as US$110.73 million (result not presented).

**Table 7:** Estimated dengue costs in Saudi Arabia during the period 2013–2017 including the correction for under-reporting.

Parameter	2013	2014	2015	2016	2017	Total 5-year Cost (2013–2017)
**Total No. cases**	12570	13083	14409	21657	15516	**77235**
**Confirmed**	10098	4572	9486	13245	8706	**46107**
**%confirmed**	80.3%	34.9%	65.8%	61.2%	56.1%	**59.7%**
**EDC (SAR)**	61219975.5	42973148.1	78758628.9	108679163.3	84904139.4	376535055.2
**EIC (SAR)**	477815066.4	142205561.4	336544410	523330203.9	209316581.3	1689211823
**ETC (SAR)**	539 035 041.9	185 178 709.5	415 303 038.9	632 009 367.2	294 220 720.7	**2 065 746 878**
**ETC (US$)**	143 742 677.8	49 380 989.2	110 747 477.0	168 535 831.3	78 458 858.84	**550 865 834.2**

Under-reporting was estimated as follows: for every reported dengue case, there are 3 actual dengue cases (i.e., expansion factor = 3). EDC: Estimated direct costs using the predictive model based on source data; EIC: estimated indirect costs using the GDP-based model; ETC: Estimated total costs; SAR: Saudi Riyal; US$: United States Dollar.

## Discussion

This is the first study investigating the economic burden of dengue fever infection in Saudi Arabia, based on local data. Its findings emphasized the substantial economic burden of dengue fever in Jeddah and surrounding areas, representing the endemic center of the country and weighing heavily on the national economy. Dengue fever costs were divided into direct and indirect costs, each analyzed using a specific method. For direct costs, we employed a bottom-up approach based on data collected from a representative sample of laboratory-confirmed cases of dengue fever from the highest endemic region in Saudi Arabia during 2016. Direct costs of the sample were modeled as a function of predictors and the predictive model was applied on national data to estimate the respective direct costs. For indirect costs we employed the Human Capital Method, which is an economic model that is used in most of COI studies and is based on the estimation of productivity lost costs by adjusting the GDP per capita [34,40].

In general, three major methods are used to estimate indirect costs in the literature, including the willingness to pay method (WTP), friction cost method (FCM), and human capital method (HCM). In the WTP, the amount which an individual is eager to pay to alleviate the burden of an illness or mortality is quantified. The FCM relies on the productivity lost due to time required to restore the original production level. In such a method, the productivity losses are probably replaceable and thus the main losses are limited to a short period, during which a sick employee could be replaced. FCM is a conservative method because job absenteeism or death are the major determinants of productivity loss. In HCM, the person is viewed as a capital investment and can be valued by his/her societal economic contribution. Therefore, in cases of dengue fever, the temporary productivity loss is estimated as the discounted present value of future earnings expected over the course of the infection, considering the assumption that future earnings are used as a proxy for future productivity ^[^^41^^]^.

Although both methods used in this study are statistically and methodologically valid, it is plausible that relying on multiple sources of data would provide more accurate national estimates. Furthermore, besides the results of estimated costs that are discussed in the following sections, data processing sheds light on the considerable lack of economic data with a questionable quality of the available data, which made the cost analysis laborious and yielded some methodological limitations. This denotes the need to integrate effective data collection and management procedures as part of disease surveillance and control strategies.

### Underestimation: A problematic aspect

For the total 15,369 patients included over the 5 years in the current study, the estimated yearly cost of dengue illness was US$110.17 million after assuming an EF of 3. Considering US dollar inflation rates in 2018 [42], our annual estimate was relatively in agreement with that of a recent systematic review that investigated the global economic burden showing an annual economic burden of US$99.25 million [21]. However, our figure was higher than the majority of other reports. A recent multinational study in Southeast Asia comprising 12 countries has reported an annual cost of US$31.15 million [43] after adjustment for underreporting. Furthermore, an earlier prospective study including eight countries in Asia and the Americas revealed an annual estimate of US$48.27 million based on the average of dengue cost during the period 2001–2005 without adjustment for underreporting [44]. More recent studies that adjusted their officially-reported numbers for unreported cases revealed annual estimates of US$50.91 million in Southern Vietnam [45], and US$61.06 million in Malaysia [36]. Only the estimates of Lim et al. [46] were greater than ours, indicating annual costs of US$153.16 million and US$155.46 million in Malaysia and Thailand, respectively. The high figures found in the present study are explained by the GDP per capita in Saudi Arabia being much higher than many other countries. Additionally, our estimates showed that a substantial proportion of indirect costs was related to productivity years loss, which is directly associated with the country’s GDP. Furthermore, the estimation of productivity years loss is directly impacted by the relatively high life expectancy and the young population of Saudi Arabia. Thus, productivity years loss accounted for 63 to 82% of the total costs in the present study.

The productivity loss contributes to the largest proportion of total costs in several other COI studies, accounting for 50–60% of the total costs [26,30,43], which is in line with our findings. On the other hand, vector control measures, which are considered as indirect costs, were reported to represent 40–72% of costs implied on healthcare systems when estimated in different studies[34,47,48]. Regarding the EF, the value used in the present study is relatively consistent with an EF of 3.79 in a Malaysian investigation that relied on merged data from the literature, expert opinion, and implementing a Delphi process [36]. However, our EF is significantly lower than that from studies published in Southeast Asia, Thailand and Cambodia (EF = 7.8–9.1) and Puerto Rico (EF = 10)[37–39].

Therefore, it is acceptable that our ETC estimate and EF are conservative, probably considering three major factors. First, disease estimates in the current study reflect the patients with a symptomatic dengue in its acute phase, which potentially affects the quality of life (QOL) of patients; however, chronic fatigue can occur post-dengue infection, especially in females, elders and patients having acute phase chills and may persist from 2 months to 2 years in 8.5%-57% of the patients [49–52]. These chronic consequences and their impact on the QOL may ultimately affect the total economic burden. However, they were not considered in the present study and hence the costs are likely to be underestimated. Second, economic calculations in the present study did not entail the costs of preventive measures, vector control, or surveillance programs, which may increase the overall costs by 20% to 43% [26,53,54]. Third, some additional costs can presumably exist, such as intangible costs on the healthcare system, the impact of dengue and its periodic epidemics in Saudi Arabia on tourism and other economic sectors that could not be elucidated in our study. These might be of a notable effect that renders the net cost underestimated.

### The potential reasons of cost variation

The variations in cost estimation may be affected by several factors. First, the discrepancy of costs between hospital types as indicated in the current study. More specifically, hospital type had a significant effect on the cumulative changes in EDC over the study period and was a significant predictor of direct costs of dengue. Similarly, in Thailand, there was a significant difference in the COI between large referral centers and community or provisional hospitals ^[^^55^^]^. The degrees and magnitudes of variation of COI should be evaluated in multiple facilities of the same type as suggested previously [56]. Second, transportation to healthcare facilities may contribute to outpatient costs [55,57]. However, the impact of transportation barriers seems to be only apparent in earlier studies in Saudi Arabia as revealed by 39% of patients [58], while recent evidence indicated no association between the distance to healthcare facilities and patient’s satisfaction [59].

### The cost-effectiveness of vaccination and other control programs

Dengue vaccination and dengue control programs may offer a cost-effective approach, with a significant disparity among developing and developed countries. In the early 1990s, Shepard and Halstead [60] found that dengue vaccine was cost-effective by saving an average of US$1,440 per disability-adjusted life year (DALY) and US$92.4/death averted in developing countries with poorly-developed healthcare systems. However, 10 other Southeast Asian country-based analyses [61] assumed vaccination costs of $10/dose in the private sector and US$0.50/dose in the public sector with an administration cost of US$3.50/dose. More recently, Zeng et al. [62]concluded that dengue vaccine has the potential to reduce direct and indirect costs per capita by 22% and 23% in Latin America and Asia, respectively. However, the work by Zeng et al. did not consider serological status prior to vaccination, which is highly recommended due to risk of severe dengue among individuals without prior exposure to the virus. Therefore, other researchers assessed the cost-effectiveness of combined serological screening and vaccination strategy, where only seropositive people would be candidate for vaccination [63–65]. Such strategy aimed at reducing DENV transmission and the related economic burden. These authors analyzed different scenarios that considered the variability, across settings, of DENV transmission rate, diagnostic performance of the serological screening test (i.e. sensitivity and specificity), the cost of vaccination and serotesting, and the value of disability-adjusted life-year. Although mitigated, findings generally indicated that cost-effectiveness of such campaigns could be expected in settings with a high transmission rate of DENV and high burden of dengue, provided that the sensitivity of the serotesting is adequate. On the other hand, a high transmission rate may downplay the added value of serotesting in such a strategy. Furthermore, one of the aforementioned models suggests that repeated serotesting would improve health benefits and may result in significant economic savings, using a return-on-investment (ROI) model, thus achieving cost-effectiveness [65].

In light of these findings, it is imperative to assess the efficacy and cost-effectiveness of dengue vaccination campaigns in Saudi Arabia, besides other preventive and surveillance programs, using local epidemiological and economic indicators, with the aim of reducing the substantial health and economic burden of the disease [63–65].

### Limitations

In this study, we evidenced some limitations that might affect the interpretation of the outcomes. Inclusion of patients in a hospital setting indicates that they experienced relatively moderate to severe symptoms and, therefore, a proportion of mildly symptomatic patients may have remained undiagnosed and escaped inclusion. Thus, despite the correction factor (EF = 3), the included sample might be unrepresentative of the whole population. Furthermore, the used EF was based on existing literature rather than real evidence from Saudi Arabia. Applying a constant EF from non-local studies would possibly be problematic given that EF may differ according to the intensity of transmission [66], transmission season [67], patients’ age [39], disease severity [68], level of access to healthcare [38], and local variations [69]. From another perspective, it is possible that the EF may be inflated relative to the actual instances when a laboratory-confirmed patient is referred from a private hospital to the public sector after a positive result has been obtained and hence may be reported twice. Moreover, the EFs of the private sector are uncertainly estimated, particularly for ambulatory cases.

The method used for indirect costs estimation is limited by the assumptions regarding DALS due to absenteeism, which may be over-estimated especially in non-severe cases. On the other hand, this method was limited by the multiple imputations required to correct missing variables. This urges the implementation of reliable data collection and management systems to enable more accurate estimations.

Drivers of higher costs, such as prolonged disabling symptoms and effects on tourism and other economic sectors that might be associated with dengue epidemics were not considered in our statistical analysis. In addition, the inability to include costs of vaccination and vector control strategies (as they were beyond the objectives of the study) would eventually add to the limiting factors.

### Future implications

Evidently, there is a need to conduct future local studies, on a larger scale, that entail a multiregional methodological approach to provide robust national evidence. This is to overcome the potential bias that may emerge from estimating the economic burden in an endemic region like Jeddah, along with the existence of substantial spatial differences across the Kingdom.

In general, future studies should be based on standardized monetary units to facilitate the comparisons between countries of different purchasing powers and the outcomes should be analyzed considering the financial structure of local healthcare systems. It is also recommended to combine medical costs with costs related to reduced QOL, tourism estimates, and vector control expenses. A comprehensive analysis of the economic aspects in the private sector is needed to help guide decision-making and policy-making, particularly in terms of employing vaccine campaigns and the development of antiviral drugs. Estimates of productivity loss could be derived via the human capital approach rather than self-reported information that can be subject to recall bias. However, more accurate assumptions are needed for cost estimations for those working in both the formal and informal sectors. Another crucial element that should be taken in mind is quantifying the cyclical variations to capture the common patterns of disease transmission to reveal accurate cost estimates during the endemic and epidemic times. This could be partially done via collecting data over multiple years.

Importantly, intensified vector control should be a critical component of health sector planning in Saudi Arabia, particularly in light of mass gatherings during Hajj and Umrah, primarily constituted from populations from Southeast and South Asia, and the possible transmission of dengue and other vector-borne neglected tropical diseases [70–71]. The nature and duration of disease burden and its economic consequences should be studied following these religious rites.

Finally, it is essential to establish future comparative analyses of the cost-effectiveness of a dengue vaccine. This should include avoiding the inherent difficulties implied by such studies as per experts’ recommendations, including the application of well-defined and cited assumptions, considering the vetted costs of vaccination and its possible side effects as well as surveillance programs aiming to monitor the safety of new vaccines, utilizing natural units to report cost-effectiveness (such as DALY, death averted, admission averted, etc.), and the estimated effects on mortality since policymakers may wrongfully expect a high mortality.

## Conclusions

Rapid urbanization, global trade, and the exceptionally great numbers of worldwide visitors during Hajj and Umrah have all placed the Kingdom of Saudi Arabia at a significant risk of introduction of several vector-borne tropical diseases. Of them, dengue fever has been established as an endemic disease for two decades and this could be emphasized in the current study. Dengue has a substantial local economic burden that costs US$110.17 million per year resulting in an average cost of US$11 947.6 per patient. Costs were highly disparate across the different types of healthcare institutions and productivity years loss accounted for approximately 80% of the total costs.

These estimates, as with other global estimates, are subject to underestimation given several factors, including the use of unstandardized expansion factors, likelihood of exclusion of the mildly-affected cases, underestimation of other symptoms and chronic complications that have a prolonged economic impact, and exclusion of vector control and other preventive activities from the analysis. In light of study limitations, there is a need to conduct future studies employing a multiregional approach, using standardized monetary units¸ quantifying cyclical patterns of the vector and health burden, and investigating the cost-effectiveness of a dengue vaccine.

## Supporting information

S1 Fig Estimated indirect costs of dengue fever in Saudi Arabia by year (2013–2017) using three different imputations for missing key variables. (TIF)
