# Supplementary figures and images for: The economic burden of dengue fever in the Kingdom of Saudi Arabia
Source: PLoS Negl Trop Dis. 2020 Nov 30;14(11):e0008847. doi: 10.1371/journal.pntd.0008847 (PMC7728199; doi:10.1371/journal.pntd.0008847)

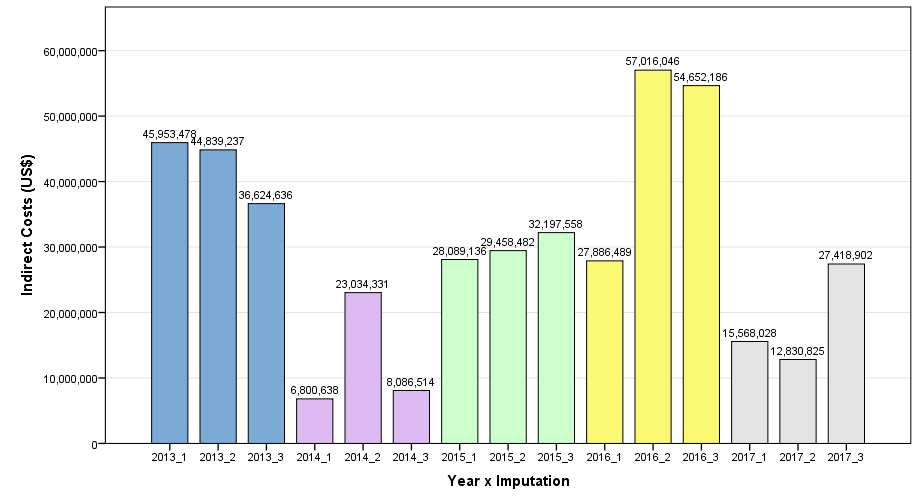

Supplement: S1 Fig — (TIF) [file pntd.0008847.s001.tif]
